# Strengths and complementarity of systematic conservation planning and Key Biodiversity Area approaches for spatial planning

**DOI:** 10.1111/cobi.14400

**Published:** 2024-10-15

**Authors:** Andy Plumptre, Jack Hayes, Daniele Baisero, Rob Rose, S. Holness, Lize von Staden, Robert J. Smith

**Affiliations:** ^1^ KBA Secretariat Cambridge UK; ^2^ Conservation Science Group, Department of Zoology Cambridge University Cambridge UK; ^3^ William and Mary University Williamsburg Virginia USA; ^4^ Centre for African Conservation Ecology & Institute for Coastal and Marine Research Nelson Mandela University Gqeberha South Africa; ^5^ SANBI Pretoria South Africa; ^6^ Durrell Institute of Conservation and Ecology University of Kent Canterbury UK

**Keywords:** Global Biodiversity Framework, Key Biodiversity Areas, Marxan, spatial planning, systematic conservation planning, áreas clave de biodiversidad, Marco Mundial de Biodiversidad, Marxan, planeación espacial, planeación sistemática de la conservación, 空间规划, 关键生物多样性区域, 系统保护规划, Marxan软件, 《全球生物多样性框架》

## Abstract

Developing biodiversity‐inclusive spatial plans at a national level is the focus of Target 1 of the Kunming–Montreal Global Biodiversity Framework (KMGBF). There are 2 general approaches to identifying areas of value for biodiversity plans: criteria‐based, such as the Key Biodiversity Areas (KBA) process, and systematic conservation planning (SCP) approaches, which apply complementarity to efficiently achieve specific quantitative targets. We examined the benefits of both approaches and considered how the KBA approach can best complement SCP. We reviewed 200 papers articles that applied SCP to real‐world data with the Marxan conservation design software. Our review showed that targets for biodiversity elements are poorly selected in many SCP publications, with more than 75% of the studies applying uniform percentage target amounts to planning elements. Uniform targets favor more widespread species and ecosystems that are likely to be more common and less important for conservation. The strengths and complementarities of KBA and SCP approaches were reviewed and we identified the elements from both approaches that should be considered for spatial planning to achieve Target 1 in the KMGBF. In particular, the global approach of KBAs (i.e., identifying sites of global significance for species or ecosystems) better complements SCP, which often has a national or subnational focus. The KMGBF will fail if conservation of globally significant sites is not targeted and these sites are not incorporated in national spatial planning.

## Graphical abstract

In order to achieve Target 1 of the Global Biodiversity Framework there is a need to develop a biodiversity‐inclusive spatial plan for a country. We explore in this paper how criteria‐based approaches, such as the Key Biodiversity Areas programme, benefits the production of such plans and how they can be used to improve Systematic Conservation Planning at national scale.

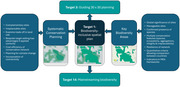

## Abstract (es)

Fortalezas y complementariedad de la planeación sistemática de la conservación y el enfoque de áreas clave de biodiversidad para la planeación espacial

**Resumen:**

El desarrollo de planes espaciales que incluyan la biodiversidad a escala nacional es el objetivo 1 del Marco Mundial de Biodiversidad Kunming‐Montreal (KMGBF). Existen dos enfoques generales para identificar áreas de valor para los planes de biodiversidad: el basado en criterios, como el proceso de Áreas Clave para la Biodiversidad (ACB), y los enfoques de planificación sistemática de la conservación (PSC), que aplican la complementariedad para alcanzar eficazmente objetivos cuantitativos específicos. Analizamos las ventajas de ambos enfoques y estudiamos cómo el enfoque ACB puede complementar mejor la PSC. Revisamos 200 artículos que aplicaban el PSC a datos del mundo real con el software de diseño de conservación Marxan. Nuestra revisión mostró que las metas para los elementos de biodiversidad están mal seleccionadas en muchas publicaciones de PSC, con más del 75% de los estudios con cantidades porcentuales uniformes de metas a los elementos de planificación. Los objetivos uniformes favorecen a las especies y ecosistemas más extendidos que probablemente son más comunes y menos importantes para la conservación. Revisamos las fortalezas y las complementariedades de los enfoques ACB y PSC e identificamos los elementos de ambos enfoques que deben considerarse en la planificación espacial para alcanzar la meta 1 del KMGBF. En concreto, el enfoque global de las ACB (es decir, la identificación de lugares de importancia mundial para las especies o los ecosistemas) complementa mejor el PSC, que suele tener un enfoque nacional o subnacional. El KMGBF fracasará si no se tiene como objetivo la conservación de los lugares de importancia mundial y si estos lugares no se incorporan a la planificación espacial nacional.

## 系统性保护规划和关键生物多样性区域方法在空间规划中的优势和互补性

**【摘要】** 在国家层面制定生物多样性包容性空间规划是《昆明‐蒙特利尔全球生物多样性框架》(Kunming‐Montreal Global Biodiversity Framework, KMGBF)目标1的重点。确定生物多样性计划中具有保护价值的区域一般有两种方法:一种是基于标准的方法, 如关键生物多样性区域(Key Biodiversity Area, KBA)流程;另一种是系统性保护规划(systematic conservation planning, SCP)方法, 它们相互补充以有效实现具体的定量目标。本研究分析了这两种方法的优势, 并考虑了KBA方法如何与SCP达到最好的互补效果。我们回顾了200篇利用Marxan保护设计软件将SCP应用于实际数据的论文。综述表明, 在许多SCP论文中, 生物多样性要素的目标都没有得到很好的选择, 超过75%的研究对规划要素采用了统一百分比的目标量。统一目标有利于分布更广泛的物种和生态系统, 但这些物种和生态系统可能更常见, 对保护的重要性也更低。我们总结了KBA和SCP方法的优势和互补性, 并确定了这两种方法中应考虑用于空间规划以实现KMGBF目标1的要素。我们特别指出, KBA的全球化方法(即确定对物种或生态系统具有全球重要性的地点)能更好地补充SCP, 而SCP的关注重点通常为国家或国家以下的尺度。如果不以保护具有全球重要性的地点为目标, 也不将这些地点纳入国家空间规划, 那么KMGBF未来很可能会失败。**【翻译:胡怡思;审校:聂永刚】**

## DEVELOPING NATIONAL SPATIAL PLANS FOR BIODIVERSITY

The Kunming–Montreal Global Biodiversity Framework (KMGBF), ratified in 2022, contains 4 goals and 23 targets to be achieved by 2030. Target 1 aims to “ensure that all areas are under participatory, integrated, and biodiversity inclusive spatial planning and/or effective management processes addressing land and sea use change, to bring the loss of areas of high biodiversity importance, including ecosystems of high ecological integrity, close to zero by 2030, while respecting the rights of indigenous peoples and local communities” (CBD, 2022a; Gurney et al., 2023). Spatial plans produced at an appropriate scale and based on data showing areas of importance for biodiversity (Plumptre et al., 2024) can be used to direct infrastructure development, agricultural expansion, and other land‐ and sea‐use changes to minimize impacts on biodiversity. These plans can also help mainstream biodiversity conservation in government planning (Target 14 of KMGBF). Conservation scientists and practitioners generally use two transparent and repeatable approaches for informing spatial planning, criteria‐based and systematic conservation planning (SCP) approaches, both of which are ideally placed to help achieve the relevant KMGBF targets but that differ in what they achieve.

### Criteria‐based approaches

Criteria‐based approaches employ criteria and thresholds to identify individual sites, such as the criteria used to identify Key Biodiversity Areas (KBAs) (sites of global significance for biodiversity). For example, a site can be a KBA if it contains 1% or more of the global population of a species published as vulnerable by the International Union for Conservation of Nature on the Red List of Threatened Species (IUCN, 2016). The 11 criteria and quantitative thresholds established for KBAs were identified through extensive consultations and testing across different taxonomic groups of species (Langhammer et al., 2018) and relate to the proportion of the global population of a species or global extent of an ecosystem at a site. These areas include criteria for species, ecosystems, and sites of high ecological integrity or irreplaceability, building on and complementing other criteria‐based approaches that have limited taxonomic scope (Plumptre et al., 2024). We focused on the KBA approach, but the comparison would also apply to other criteria‐based approaches. Although KBAs have been identified in nearly every nation, these countries have not been assessed comprehensively by applying most KBA criteria across all taxonomic groups and ecosystems. The KBA data are therefore partial and are being added to regularly. However, the sites are valuable for guiding conservation planning (see below).

### SCP approaches

SCP is an operational model for efficiently identifying and implementing the conservation of priority areas (Margules & Pressey, 2000; Moilanen et al., 2009). It consists of 3 broad stages: first, framing to identify the context and broad objectives; second, spatial conservation prioritization; third, implementation. The second stage first involves stakeholders identifying a list of biodiversity elements, known as conservation features, to be included in the planning process. Most SCP analyses then use specific software to identify an efficient set of areas in a complementarity‐based approach, such as Marxan (Ball et al., 2009), Zonation (Moilanen, 2007), C‐Plan (Pressey et al., 2009), or Prioritizr (Hanson et al., 2022).

These use different selection algorithms, but they are all based on the concepts of adequacy, representativeness, and efficiency, and they apply complementarity and connectivity (Linke et al., 2011). Adequacy involves selecting sites to ensure the long‐term persistence of the region's biodiversity. This commonly involves setting quantitative targets to specify the minimum amount of each conservation feature required (Carwardine et al., 2009). Representativeness involves choosing conservation features that represent broader biodiversity, including species, ecosystem types, ecological processes, and ecosystem services. Efficiency involves identifying networks of priority areas and applying complementarity to achieve the conservation objectives and targets while minimizing cost. Depending on the data used as the cost metric, this can range from minimizing the total area of the network to minimizing the conservation budget needed or the impacts on other sectors, such as agriculture, forestry, or fishing (Naidoo et al., 2006). Outputs of these tools have been used as a surrogate for irreplaceability of sites—the degree to which they are necessary to achieve a solution (Baisero et al., 2021; Ferrier et al., 2000; Pressey et al., 1994).

The KBA and SCP approaches are both useful in generating spatial plans but generate different outputs. Building on Smith et al. (2019), we considered how best to use them in combination, both directly and by using their underlying principles to inform the broader planning process to achieve Target 1 of the KMGBF.

## ELEMENTS TO INCORPORATE IN SPATIAL PLANS FOR BIODIVERSITY

Spatial plans will only be effective in supporting the reversal of biodiversity loss if they incorporate the relevant features needed to best represent conservation in a country and have the support and buy‐in of relevant stakeholders. With spatial planning, a map needs to be developed that incorporates the following.

### Data on a wide set of biodiversity features

Recent publications propose that the biodiversity features targeted by KBA criteria should be used to target protected and conserved area expansion (Watson et al., 2023) or when defining areas of importance for biodiversity (Plumptre et al., 2024). The KBA criteria are nested within 5 types of biodiversity features targeted by conservation efforts: threatened species and ecosystems; geographically restricted species, species assemblages, and ecosystems; ecological integrity; biological processes where species congregate; and irreplaceability. In addition, areas of connectivity or areas important for people, such as ecosystem services and sites important for national and cultural reasons, could also be incorporated (Watson et al., 2023). Failing to apply all the KBA criteria in a national KBA assessment or failing to incorporate all these features in an SCP process will lead to a less robust result. The KBAs can therefore help guide the overall objectives of SCP in a country to ensure that the planning process considers all relevant biodiversity features and sites that are globally significant for conservation.

### Accurate feature distribution data

Planning needs to ensure that the biodiversity features targeted for conservation are actually present at sites identified. KBAs require confirmation that a species or ecosystem is present before they can be published in the World Database of KBAs (BirdLife International, 2024). SCP often uses modeled species distributions or maps of ecosystems, but it also needs to be confirmed that these elements are present at sites selected through the planning process.

### Conserves enough of each feature so it can persist but not so much that it is not feasible to implement

The KBA criteria aim to identify sites where sufficient reproductive units occur to ensure the continued persistence of the species that trigger KBA status. However, not all KBAs may be needed in a final spatial plan. The proportion of the global population of a species or global extent of an ecosystem is estimated for KBAs, and this information is useful for deciding how to incorporate KBAs in SCP. The SCP processes identify efficient solutions given a set of objectives and targets for each biodiversity element, but the solution needs to be interrogated to assess the viability of selected sites. Both processes can therefore inform the other.

## KBA PLANNING IN AN SCP CONTEXT

The framing process is a fundamental component of SCP because it involves translating the conservation context into a set of broad objectives (Groves & Game, 2016). Even if two analyses are based on exactly the same region and biodiversity data sets, their results will differ if contrasting objectives lead to stakeholders choosing different targets and cost–benefit metrics. A key component and strength of the SCP process is working with stakeholders to decide and agree on a set of common planning objectives because these will have significant impacts on the final results. Understanding this makes it much easier to interpret the different ways in which KBAs can be used to inform policy and action. In some situations, the context leads to setting 100% targets for every KBA, ensuring that each site is locked into the SCP result. For example, Mozambique incorporated KBAs into their National Territorial Plan and legislation to give every site higher protection. By doing so, they effectively set 100% targets for KBAs. Similarly, an SCP analysis for Uganda locked into the analysis all KBAs to ensure they were part of the plan; complementarity was used to identify additional areas needed to conserve target species and ecosystems that were generally of national importance (Plumptre et al., 2019).

However, there are other contexts in which stakeholders might set lower targets because costs of the associated actions are higher or the available resources are lower. For example, a project might select a subset of KBAs for further investment to improve their management effectiveness, whereas others may be deemed safe for now given limited resources. In such a case, the prioritization stage could use data on each site's biodiversity and the extent to which it fills representation gaps in the existing network, as well as the management costs, threats to sites, and likelihood of success (Gardner et al., 2015; Joseph et al., 2009). How KBAs are used in the SCP is context dependent, based on the biodiversity value of the sites and the objectives of the planning process.

## USING THE KBA STANDARD AND KBAs IN SCP

The global standard for the identification of KBAs (KBA Standard [IUCN, 2016]) describes the rationale and method for identifying KBAs. It builds on previous criteria‐based approaches and was codeveloped by many experts from across the world. This means the KBA Standard provides a number of important insights for informing spatial planning, three of which are particularly relevant for SCP.

### KBAs as globally significant sites

SCP generally involves stakeholders producing a list of important conservation features and setting quantitative targets for each for their inclusion in a network of priority areas (Margules & Pressey, 2000). Without guidance, there is a tendency for local stakeholders to base these decisions on the local or national status of a species or ecosystem, especially for features that are culturally significant or charismatic. For example, impala (*Aepyceros melampus*), a globally least concern species, was a priority species in Uganda because it gives its name to the country's capital city Kampala and was listed on the national red list as endangered because of a declining national population (WCS, 2016). Incorporating national priorities in spatial plans is clearly sensible but must not be done at the expense of global or regional priorities. SCP analyses often use country priority species lists when identifying what biodiversity to include, and these lists can be dominated by national priorities or charismatic species, rather than species that are globally threatened or endemic to the country (e.g., Lim et al., 2023).

In contrast, KBAs contain globally or regionally significant populations of a species, globally or regionally significant extent of an ecosystem, or sites of outstanding ecological integrity or irreplaceability. As such, they complement nationally important sites, identifying sites for which countries are more likely to have a global responsibility for the conservation of a species or ecosystem. The fact that the KBA criteria use a standard approach that is transparent and repeatable (quantitative criteria and specific thresholds) also means they are comparable among countries and regions (Plumptre et al., 2024). This creates two important reasons for including KBAs in SCP analyses. The first is that KBAs provide a foundation, flagging sites of global importance in a world where priorities are often shifting. The second is that KBAs are used by the international community, the private sector, and donors to inform cross‐national monitoring and decision‐making. For example, the “proportion of KBAs covered by protected areas” is an indicator for the CBD KMGBF and the UN Sustainable Development Goals 14 and 15 (CBD, 2022b).

### KBAs as important sources of data

SCP and KBA assessments commonly use data on spatial distributions of species derived from range maps or habitat maps. All such maps have commission and omission errors, so it is possible that some priority areas identified in SCP analyses do not contain the species for which they were selected (Rondinini et al., 2006). It is important to collect available point location data or carry out field surveys to identify whether the species are present in each priority site (Johnson et al., 2023). There are obvious benefits in using more accurate species distribution data as part of SCP and KBA analyses. However, the process of proposing a site as a KBA involves determining that the trigger element is definitely present at a site with sufficient numbers for reproduction and meets the relevant KBA criteria (KBA Standards & Appeals Committee, 2022). A species or ecosystem that triggers a KBA contains more than a certain threshold of the global population or global extent of the ecosystem at the site. These values of the proportion of the global population or extent of ecosystem are also valuable in guiding whether to invest in conserving a KBA or not.

KBAs continue to be identified as new data become available in countries around the world. Where KBAs have been relatively comprehensively assessed, applying most criteria across multiple taxonomic groups and ecosystems, then clearly the KBAs identified will be more complete and therefore more useful for SCP. However, we believe there is still value in knowing a few of the globally important sites and using these in SCP, updating the SCP as more data are obtained and as more KBAs are identified.

### KBAs and the ability to maintain populations at a site

Another problem with relying on modeled distribution maps in spatial planning is knowing whether a site contains a population of each species that can continue reproducing. SCP analyses often implicitly account for this by setting higher targets for species found at lower densities (Solomon et al., 2003) or including data population viability data (Carroll et al., 2003), but this adds further uncertainty to the modeling process. The KBA approach helps tackle this, with its focus on only giving KBA status to sites containing populations of trigger species with sufficient reproductive units to maintain a population. Often the ways these numbers are assessed include evidence the species is being maintained at a site over several years. Including KBAs based on aggregations of species is also important because these contain important breeding sites, hibernacula, migratory feeding or roosting sites, and other congregations of individual animals.

## ASSESSING HOW KBAs CAN GUIDE TARGET SETTING IN SCP

As globally significant sites for biodiversity, KBAs provide an important source of biodiversity data and account for species reproduction, and there is obvious scope for including them in SCP analyses. In particular, setting objectives for all the biodiversity features targeted by the KBA approach will help deliver a more robust spatial plan when applying an SCP process. Including KBA data can ensure that SCP analyses identify networks of sites that are more representative, by including data on a wider range of biodiversity, and the KBA approach has the potential to help guide target setting.

Pressey and Bottrill (2009) provide 11 useful steps that are needed for conservation planning: scoping and costing the planning process (Step 1); identifying and involving stakeholders (2); describing the context for conservation areas (3); identifying conservation goals (4); collecting data on socioeconomic variables and threats (5); collecting data on biodiversity and natural features (6); setting conservation objectives (7); reviewing current achievement objectives (8); selecting additional conservation areas (9); applying conservation action to these areas (10); and maintaining and monitoring conservation areas (11). Many of these steps will be similar when undertaking a comprehensive assessment of KBAs or making an SCP plan in a country (Steps 1–3, 5, 8, and 10). Table 1 provides a summary of the different ways the KBA and SCP approaches are implemented when applying Steps 4, 6 7, 9, and 11 and summarizes the ways KBAs can contribute to spatial planning in these steps.

**TABLE 1:** Comparison of what systematic conservation planning (SCP) and Key Biodiversity Area (KBA) approaches bring to conservation planning for specific objectives where KBAs are relevant.

Objective	KBA approach	SCP approach
*Identifying conservation goals (Step 4* ^a^ *)*		
Ability to incorporate different conservation objectives for biodiversity	Criteria ensure that most features of biodiversity used in conservation are considered: threatened elements, geographic restriction, ecological integrity, biological processes, and irreplaceability	Can be improved by considering the KBA criteria and incorporating the various biodiversity features in the analysis; can also incorporate many other objectives, such as connectivity, ecosystem services, and other land uses, weighting the values of each
Identify sites of global and regional significance for individual species	Criteria use thresholds that relate to the global extent of a species, ecosystem, or global significance of a site of ecological integrity	Does not automatically prioritize globally or regionally important sites (unless the study region is global or regional); users specifically incorporate this in the planning process by setting targets appropriately; KBAs identified in country can be used to identify such sites
Identify the most irreplaceable sites	If KBA criterion E is applied, irreplaceable sites are identified for a set of fixed targets for species as described in the KBA standard; criterion E uses an SCP approach to identify sites	Can identify irreplaceable sites for any set of biodiversity elements and flexible targets; estimates of irreplaceability are a standard output of most SCP analyses
Examining trade‐offs with other land uses	Sites are globally significant and can be used to inform potential no‐go areas for other land uses; data on proportion of global population and extent of a biodiversity element at a site can inform options for no‐go areas	Can specifically be used to analyze trade‐offs in options for conservation and land‐use planning by incorporating other land uses as costs in the analysis
*Collecting data on biodiversity and other natural features (Step 6)*		
Data types used	Local presence and global distribution of species, ecosystems, and sites of ecological integrity or irreplaceability	Species, ecosystems, ecosystem processes, and other land uses (agriculture, mining, urban, etc.); where data are scarce, sometimes proxies for biodiversity are used, such as biophysical layers (e.g., Ban et al., 2009)
Implementation with limited data in country	Can start identifying KBAs from initial set of biodiversity elements so can start conserving sites before comprehensive data on all elements are obtained. Not all KBAs will be identified though until all data are compiled and analyzed	Requires comprehensive data on many biodiversity elements that span the country before the analysis can be made; a more efficient result will be produced by compiling data than identifying KBAs in a piecemeal fashion
Implementation with good country data but limited global data	Cannot identify KBA if global data on biodiversity element are limited	Can run assessment on comprehensive local data where global data are not known; tends to focus on national rather than global priorities
Effect of increasing data knowledge or adding biodiversity elements	Can easily identify new KBAs or add biodiversity elements to existing KBAs one at a time, modifying site boundaries if necessary	New data can change the solution and require replanning; may lead to loss of sites, replaced by more efficient ones elsewhere
Ability to quickly update with new information	Much quicker to identify and implement conservation at sites	Takes time to incorporate new data in existing SCP plans
Certainty that the biodiversity elements are present at a site	Identification requires evidence that a species or ecosystem is present at a site before the KBA is confirmed	Many analyses use modeled species distributions or predicted habitat maps (64% in our review); good practice ensures species and ecosystem presence at a site, and importance for that site in the final solution is assessed once identified
*Setting conservation targets (Step 7)*		
Ability to set targets amounts for a species/ecosystem	Specific thresholds set in the criteria; criterion E partially allows for individually defined targets based on functional outcomes under subtargets that aim to ensure long‐term viability of the biodiversity	Planners can set targets and assess how results change by changing target levels; careful thinking is needed when setting targets (as we demonstrate below)
Aim to set targets for some or all species and ecosystems	Identified for species, ecosystems, and ecological integrity in a piecemeal way where data are available; widespread, unthreatened species often not KBA triggers and sites are not identified for their conservation	Aims to incorporate all biodiversity elements and use complementarity to find an efficient solution to conserve them all
Identify sites for important aggregations, refugia, or spawning sites for species	Criteria exist (D1, D2, and D3) for explicit aggregations of species for breeding or migration, sites of refugia for species in times of environmental stress, and sites important for breeding or spawning	Harder to incorporate sites important for aggregations, as refugia, and for spawning with one target for a species but can specify particular aggregation sites for a species and lock them in or treat as a separate target; can use KBAs triggered by criterion D to guide this
Ability to plan for future climate change	Only identified based on current distributions of biodiversity elements and are not identified under predicted future distributions	Modeled species and ecosystem distributions predicted under climate change can be incorporated in SCP to plan for now and the future (Alagador & Cerdeira, 2020)
Ability to incorporate ecosystem services in conservation plan	Identify important sites for biodiversity and can be important for ecosystem services but do not target ecosystem services in the criteria	Can incorporate ecosystem services in the plans, as well as species and ecosystems (Villarreal‐Rosas et al., 2020)
Ability to incorporate connectivity between sites	Criteria do not consider connectivity except where there are aggregations of species, on migratory routes for instance	Can specifically incorporate planning for connectivity, particularly tools, such as Marxan Connect
*Identifying additional conservation areas (Step 9)*		
Ensuring what is needed for conservation is identified	Criteria ensure most biodiversity features used in conservation are considered when identifying sites, including threatened elements, geographic restriction, ecological integrity, biological processes, and irreplaceability	Can incorporate whatever is considered important but often SCP plans omit key elements because they were not considered; KBA biodiversity features can be used to guide thinking about what needs to be included in plans (Plumptre et al., 2024)
Minimizing costs and maximizing efficiency	Costs not specifically incorporated—some redundancy as a result and more costly outcomes if all sites are conserved	Aims to identify an efficient and often cost‐effective solution to conserve all targets for biodiversity elements
Scenario analysis and comparison of options	Site is either a KBA or it is not; how KBAs are then used in planning is determined by the planning objectives; scenarios can be developed that vary the degree to which KBAs are locked into a plan	Allows the examination of scenarios and trade‐offs in possible options for conservation to identify the most harmonious solution with a group of stakeholders
Resilient to loss of sites	Can identify multiple sites; all may not be required to ensure conservation of the biodiversity element triggering KBA status; with loss of protected areas increasing globally, it is useful to know where you have flexibility	Can identify an efficient solution, but with loss of sites, it may need to be reanalyzed; if there is concern that it will take time to conserve a final planning solution, then redundancy should be built into the targets for the conservation features
*Maintaining and monitoring conservation areas (Step 11)*		
Monitoring sites	KBA program has specific monitoring protocol for all sites identified that assesses the status of KBA trigger elements, the pressures they face, and the conservation actions being applied; can provide a standardized monitoring of sites and measure the status of KBAs	Monitoring implementation of an SCP plan and the conservation of sites identified needs to be built into all SCP outputs; if sites are lost, there may be a need to update the plan or at least assess what biodiversity elements required that site to meet their targets and identify alternative sites to target; KBA data may be useful here to help identify suitable alternatives
Comparison of sites between countries or regions: a benefit for global monitoring (e.g., CBD and SDGs)	Using of a standard set of quantitative criteria and thresholds enables the number, extent, and status of sites to be compared between countries	Use of specific targets for conservation features that will differ between studies makes comparison difficult between countries; a comparison of the elements used in the plans can be compared
Sites recognized in global policies	KBA Global Standard developed and recognized by the global conservation community; sites are recognized in safeguarding policies of finance and private sector as indicators and sites of importance for biodiversity in multilateral agreements	Different targets and SCP approaches are used at national and subnational levels, which makes it harder to develop one‐off categorizations of sites for treatment in global‐level policies; SCPs at national level therefore tend to reflect national priorities and policies
Spatial planning in Target 1 of KMGBF	Percentage of spatial plans that use KBAs is a specific indicator for Target 1 of the KMGBF; use of KBAs as input data for the SCP process strengthens final outcome, ensuring globally and regionally significant sites are incorporated in the plan	Target 1 of KMGBF encourages spatial planning, and ideally all countries would use SCP to identify the most efficient places to conserve as much biodiversity as possible while minimizing impacts for national development; borrowing from KBA criteria to ensure all biodiversity features are conserved ensures plans contribute to halting extinction and reducing biodiversity loss (KMGBF goal A)

^a^ We use the conservation planning steps outlined by Pressey and Bottrill (2009) to group specific actions.

To estimate the potential for KBAs to provide such benefits, we reviewed the scientific literature to investigate the types of biodiversity data used in SCP analyses and the extent to which broad target‐setting approaches have been applied. We restricted our analyses to research that used Marxan or Marxan with Zones (or prioritzr applying a Marxan‐like analysis), the most widely used SCP software package, and identified articles published in the literature through Science Direct (https://www.sciencedirect.com), Wiley Online Library (https://onlinelibrary.wiley.com), and Web of Science (https://www.webofscience.com). Our search terms were *Marxan* and “*systematic conservation planning*.” We also searched for websites with reports of studies that have been applying results of Marxan analyses (e.g., https://marxansolutions.org/community). We compiled papers until we reached 200 papers and reports that used specific real‐world data. We omitted papers that were about the general design or use of the software. Of these, 104 were planning for terrestrial regions, 67 marine regions, and 29 for both terrestrial and aquatic (marine or freshwater), and they were published from 2004 to 2023.

### Biodiversity representation

For each of the 200 studies, we determined whether they used data on ecosystems, species, or other elements, where *other* includes ecosystem services, biophysical complexity, measures of connectivity, and climate change (Appendix S1). Most studies (71%) used species data, often with combinations of ecosystem (54%) and other (29% of studies) data types. Terrestrial studies tended to use more species data (81%) than marine (61%) and terrestrial and aquatic (59%) studies. Quite a few studies only used species data (34%). For studies in which species data were used, 64% used expert maps or species distribution models to identify habitat, 15% used IUCN range maps, 13% used point data, and 8% used a combination of data types for species. Our study found that 17% of studies only used ecosystem data in their analyses. Of those studies that did include species data, many used sources that could have contained commission errors; IUCN range maps and even species distribution models will predict that a species is present when in fact it is absent (Rondinini et al., 2011). Only one study considered ecological integrity and none incorporated sites where species aggregated, such as migratory feeding sites or breeding congregations. Once again, including KBA data and targeting the biodiversity features used to identify KBAs would help resolve these limitations by identifying priority areas where the relevant species are known to occur.

### Setting conservation targets

Setting appropriate targets is extremely important for target‐based spatial prioritization, and the methodology used can “have far‐reaching implications and will have to be defended” (Ardron et al., 2010). Levin et al. (2015) advocated conducting sensitivity analyses of the results of SCP by varying the targets systematically. Similarly, Carwardine et al. (2009) warn about the importance of correctly setting and interpreting SCP targets and solutions. Ideally, targets should be based on both the conservation context, as defined during the SCP framing stage, and the SCP principles of adequacy and representativeness. To investigate this, we defined 5 approaches for setting targets and categorized the approach used in our sample of 200 SCP studies: uniform, grouped, variable formula, context driven, and functional targets.

A uniform targets approach assigns the same percentage target of the distribution for all the species, ecosystems, and other features used in the analysis. For example, for each conservation feature, the target would be 10% of its distribution.

A grouped targets approach is similar to a uniform targets approach except that different percentages are given to groups of species or ecosystems. For example, threatened species may have a target of 30% of their distribution, whereas nonthreatened species have a target of 10%.

In a variable‐formula targets approach, different percentage targets are applied to each biodiversity element based on a formula. For example, the target assigned to a species may be in proportion to its global range area, with larger target percentages set for species with smaller ranges (e.g., Hanson et al., 2020; Harris & Holness, 2023; Rodrigues et al., 2004; Schuster et al., 2023).

In a context‐driven approach, targets are set based on a specific conservation context, rather than to achieve a certain biological outcome. For example, targets are set to achieve the best realistic solution for a specific project with a set of management aims and associated budget, rather than what is required to fully conserve all the species or ecosystems in the long term.

Functional targets are set individually for each biodiversity element to achieve a species‐specific or ecosystem‐specific conservation outcome based on specific ecological knowledge.

**FIGURE 1:**
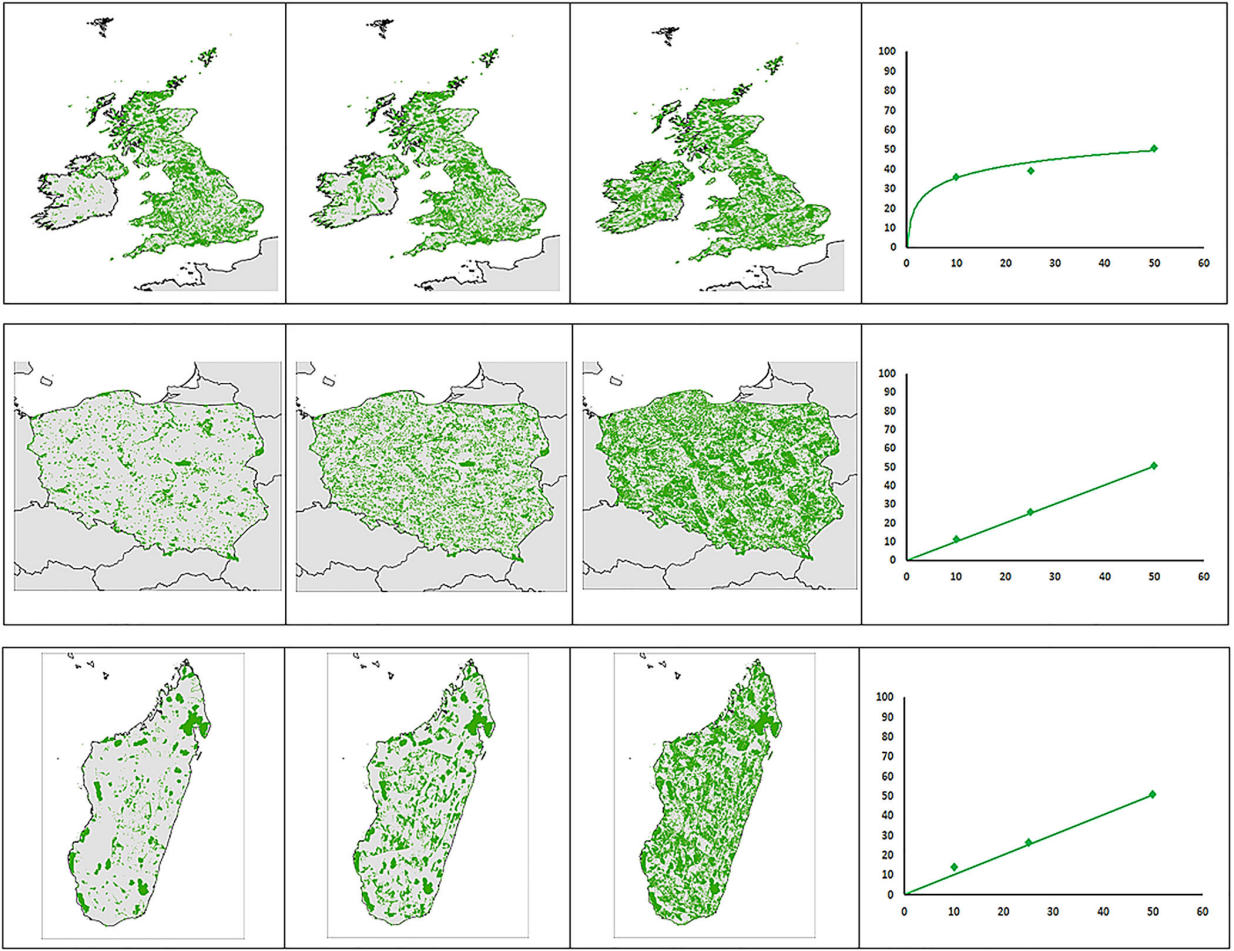
Comparison of systematic conservation planning solutions identified with a Marxan approach in Prioritizr for the British Isles (top), Poland (middle), and Madagascar (bottom). Maps show the solution for targets of 10% (left), 25% (center), and 50% (right). The change in percentage of total area (*y*‐axis) is plotted on the right for each country against the percentage target (*x*‐axis). Methods used in this analysis are in Appendix S2.

Our results showed that uniform targets (34%) and grouped targets (43%) dominated the target‐setting approaches across our sample of 200 studies (Table 2). This is a concern because uniform targets and, to a lesser extent, grouped targets increase the representation of the conservation features with the largest distributions. These targets generally favor the more common species over rarer and geographically restricted species (Figure 1). This is particularly problematic for analyses with features that do not overlap, such as ecosystem types, where complementarity is not applied. In both situations, large parts of the priority areas identified in the SCP analysis will be driven by the presence of a few widely distributed features, many of which are of low conservation concern (Vimal et al., 2011). The variable‐formula targets (4%) help address this problem by reducing targets for the most widespread features, but even studies that set a minimum target (e.g., 10%) could result in choosing large areas based on the presence of these common species. It is likely, however, that many future SCP analyses will continue to use uniform, grouped, and variable‐formula target setting approaches because the alternatives are much more data intensive, requiring more knowledge about each species and ecosystem. In such cases, we recommend achieving representation goals by setting lower generic targets and by including KBAs as conservation features to ensure that known sites of globally important biodiversity are also selected (Kirkman et al., 2019; Kullberg et al., 2019). Only 14% of the studies reviewed used functional targets, setting explicit targets for each biodiversity feature to ensure its long‐term conservation.

**TABLE 2:** The percentage of 200 published analyses that applied each of the 5 approaches used to identify target values for biodiversity elements in the Marxan analysis detailed in the publication.

Realm	Uniform (%)	Grouped (%)	Variable formula (%)	Context driven (%)	Functional (%)
Terrestrial	31.7	37.5	3.9	5.8	21.2
Marine	34.3	49.3	6.0	4.5	6.0
Terrestrial and aquatic (freshwater or marine)	41.4	44.8	0.0	6.9	6.9
All studies	34.0	42.5	4.0	5.5	14.0

## SCP INFORMING KBA IDENTIFICATION

When applying criterion E for KBAs, SCP is used to identify sites that are irreplaceable. Compilation of the data needed to make an SCP analysis is time consuming; therefore, once it is done, it would make sense to capitalize on the availability of the data to identify KBAs based on this criterion. South Africa has recently identified 205 new KBAs that trigger criterion E, made possible because they undertook a national SCP. An SCP analysis may also identify likely areas in a country that may be useful for applying other KBA criteria, particularly those that identify sites for assemblages of geographically restricted species (criteria B2 and B3).

## NATIONAL SPATIAL PLANNING WITH SCP AND KBAs

Target 1 of the KMGBF aims to achieve participatory, integrated, biodiversity‐inclusive spatial plans in all countries by 2030. What should these spatial plans include and where can SCP and KBAs contribute? Resolution 081 passed at the World Conservation Congress in 2021 encouraged governments to make comprehensive spatial plans for biodiversity and incorporate these in their CBD National Biodiversity Strategy and Action Plans. It also encouraged donors and the conservation community to support national planning processes, including the identification of KBAs, use of SCP, and identification of areas where connectivity should be maintained or restored (IUCN, 2020). Below, we summarize some of the key components that should be in these national spatial plans and show how adopting both the KBA approach and SCP would strengthen fundamental elements of national planning processes.

### Involve a range of stakeholders

The success of any planning process depends on the effective inclusion and participation of relevant stakeholders, both of which benefit from adopting elements of SCP and the KBA approach. The framing stage of SCP includes a specific step for identifying stakeholders who then work together to develop the broad planning objectives and the specifics of the spatial conservation prioritization process (Groves & Game, 2016). This helps ensure that the needs and values of marginalized groups are accounted for in the planning process while also producing results that account for broader government and societal priorities (Game et al., 2011). The KBA methodology also provides guidance on including relevant stakeholders in both the identification and delineation of KBAs (KBA Standards & Appeals Committee, 2022). This is particularly important for ensuring that a wide range of taxonomic and conservation experts are included in the process. Being clear about the desired outcomes is key to the planning process and will determine the outcome. SCP tends to produce fine‐scaled and efficient sites, whereas the KBA approach can help identify larger areas where globally significant populations of species or extents of ecosystems occur where the persistence of a species is likely.

### Identify sites that have global, regional, and national importance for biodiversity

Avery et al. (1995) proposed that conservation should focus on global, regional, and national priorities but conservation is often driven by interests at a national level. Spatial plans developed for a country may choose to focus on what is considered nationally important (e.g., species or ecosystems in decline and threatened at a national level), which can result in omitting species or ecosystems with regional or global significance but relatively widespread at a national level (Lim et al., 2023). KBAs are sites of global or regional significance for species or ecosystems because they are identified in reference to the global population or global ecosystem extent. They, therefore, provide a useful input in spatial planning to ensure that globally significant sites are incorporated.

### Incorporate all biodiversity features targeted by the KBA approach

National‐level SCP analyses generally seek to represent biodiversity by including data on broad biodiversity elements, such as ecosystem types, and a set of important species. Setting targets for the 5 biodiversity features used in the KBA approach (threatened species and ecosystems; geographically restricted species; species assemblages and ecosystems; ecological integrity; biological processes where species congregate; and irreplaceability) will ensure SCP analyses are more comprehensive and inclusive in what they aim to conserve. For example, South Africa has implemented one of the most comprehensive and effective SCP processes in any country (Pressey et al., 2003), but a recent KBA assessment identified sites important for geographically restricted species that were not an objective considered in the SCP process were not assessed as threatened on the IUCN Red List (S. Holness, personal observation).

### Building redundancy into SCP outputs

Despite efforts to conserve biodiversity, there is loss and degradation of protected areas (PADDD events [Mascia & Pailler, 2011; Symes et al., 2016]), and the impacts of climate change and other anthropogenic stressors will lead to sites no longer conserving the biodiversity for which they were established. It may not be possible to conserve the whole output of an SCP quickly, with the result that some sites may be lost before they are conserved (Pressey et al., 2013). It is therefore important to build redundancy into national conservation plans so that enough of each conservation feature continues to be adequately represented, where possible, in a number of different sites. Identification of KBAs in a country may identify several sites that conserve the same species or ecosystem and as a result may create some redundancy that would not necessarily be identified in an SCP. Setting objectives in SCP that build in some redundancy is advisable given the changes being observed.

### Identify the connectivity required to link critical populations of species

Sites can be connected for several reasons. These include the maintenance of populations of species that are at low numbers at any one site and need corridors to maintain genetic variability and the maintenance of connectivity that may allow migration under changes in climate so that species can colonize elsewhere (Hilty et al., 2020). Historically, SCP and KBAs have had limitations when identifying areas of connectivity, although recent advances in SCP and the development of Marxan Connect are creating tools that can incorporate connectivity planning (Daigle et al., 2019; Pouzols & Moilanen, 2014). Although KBA identification does not assess sites for their connectivity (apart from aggregations at migratory stopover sites), the expert consultation required for KBA identification and the knowledge about the amounts of a species at a site could help with planning where connectivity is needed. If sufficient numbers occur at a site to be viable, connectivity may not be needed.

### Identify sites important for ecosystem services and other values

Not all conservation features in conservation planning will be for species and ecosystems. Some sites will have cultural values and others will be important for ecosystem services (Neugarten et al., 2024). Even sites with low importance for biodiversity may be significant for other reasons, such as raising revenue for biodiversity conservation nationally. Usually, these values are realized at a national level, although some ecosystem services also have regional or global values, such as carbon sequestration. SCP can incorporate these values if specified correctly, although setting targets for ecosystem services may not be simple because much will be determined by how the services are measured at different sites (Neugarten et al., 2018). KBAs are not identified using ecosystem service or cultural value criteria because their focus is on areas of significance for biodiversity, not their significance to people. However, there is guidance on how ecosystem services can be assessed at the sites once identified that are applicable to any site (Neugarten et al., 2018).

## CONCLUSION

It is hoped that most countries will develop a national biodiversity inclusive spatial plan to achieve Target 1 of the KMGBF by 2030. The KBAs and SCP approaches can provide highly relevant inputs when developing national spatial plans, incorporating global, regional, and national priorities and accounting for biodiversity, connectivity, ecosystem services, and cultural values. In particular, we argue that using a combination of these 2 approaches creates synergies that provide a number of broader benefits. In general, the implementation of the results will be iterative, adding sites over time to meet overall objectives. However, underpinning this expansion process with the principles of the KBA and SCP approaches will help ensure their efficiency and effectiveness, as well as achieve the national spatial planning outcomes outlined in KMGBF Target 1 and the broader goal of halting biodiversity loss.

## AUTHOR CONTRIBUTIONS

Andy Plumptre and Daniele Baisero came up with the idea of the analysis and led the writing of the paper. Jack Hayes compiled the literature review and analysis of the 3 countries and contributed to writing the paper. S. Holness, Lize von Staden, Rob Rose, and Robert J. Smith contributed to the analyses and writing of the paper.

## Supporting information



Supporting Information

Appendix S2 Methods used to make analysis presented in Figure 1
