# Supplementary material for: Strengths and complementarity of systematic conservation planning and Key Biodiversity Area approaches for spatial planning
Source: Conserv Biol. 2024 Oct 15;39(2):e14400. doi: 10.1111/cobi.14400 (PMC11959315; doi:10.1111/cobi.14400)
Supplement: Supplementary file 2 — Appendix S2 Methods used to make analysis presented in Figure 1 [file COBI-39-e14400-s002.docx]

**Supporting information**

**Appendix S2 Methods used to make analysis presented in Figure 1**

*Applying Marxan with Uniform targets to species range data from three countries.*

This analysis was based on data that were compiled for three countries (Poland, British Isles and Madagascar) as part of an update to a global assessment of irreplaceability of species (Baisero et al. 2021) using species with polygon range data (Table S1) in the IUCN Red List of Threatened Species (IUCN 2022). To demonstrate the outcomes of a crude SCP analysis we ran a prioritisation analysis using the *Prioritizr R* package (Hanson et al. 2022) on the range data in hexagon cells of 5.6 km^2^ and analysed the solutions obtained by applying a uniform target of 10%, 25% and 50% to all species. *Prioritizr* uses mixed integer linear programming to identify the most efficient solution using complementarity, unlike Marxan which uses a simulated annealing heuristic (Moilanen et al. 2009) to estimate the solution (Marxan usually requires multiple runs of an analysis to identify a good solution), but they can provide similar results if Marxan is run with many replications (Beyer et al. 2016). We assessed these solutions generated through *Prioritizr* comparing the patterns of cells selected and the area of land selected in each study area. The analysis approach uses uniform percentage targets, as used by 34% of studies we evaluated and IUCN range data for the species as used by 15% of studies. We make these analyses to demonstrate how the results are influenced by crude targets and underlying data such as these and do not recommend that this approach is used.

The number of species with polygon range data, the median range area (km^2^) and the percentage of the country’s total area covered by a median range obtained from the IUCN Red List.

| **Property** | **British Isles** | **Poland** | **Madagascar** |
| --- | --- | --- | --- |
| Number of species | 1,359 | 1,280 | 4,153 |
| Median range area (km^2^) | 58,043 | 287,861 | 22,688 |
| Percentage area of country covered by a median range. | 17.74 | 93.06 | 4.06 |

Comparison of areas selected from the results show different responses which are dependent on the number of species and their general distribution within a study area (Figure 1). In the British Isles a 10% target selects much of England, Scotland and Wales because the range areas for many species are relatively large (Median range area is 17.7% of the country - Table S1) but also vary in where they are located with differences between north and south as well as east-west. Therefore a 10% target for each species selects a large area of land across all species. Increasing the target threshold to 25% and 50% does not change the overall result much, nor increase the area greatly as a result, except for Ireland which has fewer species, many of which are common to the British Isles. Poland has fewer and very widespread species with the median range area covering more than 90% of the country. Here the area identified follows very closely with the percentage target selected (Figure 1) because there is not a great difference in distribution of the species across the country. In Madagascar, which has many more species than the two European study areas, and where the species ranges are small (median range is 4.1% of the country area), there is a lot of overlap in the small-ranged species and the area identified increases in step with the percentage target. These results show that for Madagascar and Poland in particular, that it is the most widespread species that determine the area of the final solution, and this would be true even if it is only one species that is widespread.

**References**

Baisero, D., Schuster, R. and Plumptre, A.J. (2021) Redefining and Mapping Global Irreplaceability. *Conservation Biology*. 36:e13806 https://doi.org/10.1111/cobi.13806

Beyer, H.L., Dujardin, Y., Watts, M.E., & Possingham, H.P. (2016) Solving conservation planning problems with integer linear programming. *Ecological Modelling,* 328, 14-22. https://doi.org/10.1016/j.ecolmodel.2016.02.005

Hanson, J.O., Schuster, R., Morrell, N., Strimas-Mackey, M., Edwards, B.P.M., Watts, M.E., Arcese, P., Bennett, J., & Possingham, H.P. (2022) prioritizr: Systematic Conservation Prioritization in R. R package version 7.2.2. Available at https://CRAN.R-project.org/package=prioritizr

IUCN. (2022) *The IUCN Red List of Threatened Species. Version 2022-1*. https://www.iucnredlist.org

Moilanen, A., Wilson, K. A., & Possingham, H. P. (2009). *Spatial conservation prioritization*. Oxford, UK: Oxford University Press.
